# Association of Auricular Reflective Points and Status of Type 2 Diabetes Mellitus: A Matched Case-Control Study

**DOI:** 10.1155/2015/981563

**Published:** 2015-05-19

**Authors:** Lorna Kwai-Ping Suen, Chao Hsing Yeh, Jojo Yee Mei Kwan, Paul Hong Lee, Grace Sau Ping Yeung, Esther C. Y. Wong, Billie C. Lau, Samuel Chi Hung Tsang, Alice Siu Ping Cheung, Vincent Tok Fai Yeung

**Affiliations:** ^1^School of Nursing, The Hong Kong Polytechnic University, Hung Hom, Hong Kong; ^2^School of Nursing, University of Pittsburgh, Pittsburgh, PA 15261, USA; ^3^Centre for Diabetes Education & Management, Our Lady of Maryknoll Hospital, Hong Kong; ^4^Our Lady of Maryknoll Hospital, Hong Kong; ^5^Evangelical Lutheran Church Social Service-Hong Kong (ELCHK), Shatin Multi-Service Center for the Elderly, Lek Yuen Estate, Shatin, Hong Kong; ^6^Baptist Hospital, Hong Kong; ^7^Department of Medicine & Geriatrics, Our Lady of Maryknoll Hospital, Hong Kong

## Abstract

The reflexive property of the ear can cause various physical attributes to appear on the auricle in the presence of bodily disorders. The association of auricular signals (presence or absence of discoloration, marks after pressing, tenderness, and electrical resistance) and diabetes mellitus (DM) should be further investigated because auricular diagnosis is an objective, painless, and noninvasive method that provides rapid access to information. A matched case-control study on 282 subjects was conducted. Cases (*n* = 141) were defined as those diagnosed with type 2 DM (T2DM). Every subject in the case group was matched with the control by age and gender. Ear diagnosis was conducted in three aspects: inspection, electrical detection, and tenderness testing. Results suggest that the tenderness and electrical conductivity of some auricular points, including “pancreas and gallbladder,” “endocrine,” “kidney,” “lower tragus,” “heart,” and “eyes,” were associated with T2DM status in Chinese population. In the subgroup analyses, certain auricular signals were also associated with glycemic control, disease duration, and related complications. Auricular diagnosis could be considered as a screening method for vulnerable populations with T2DM risk. Thus, appropriate interventions can be implemented to prevent or delay the progression of T2DM.

## 1. Introduction

The ear was originally mentioned in the earliest Chinese medical book* Yellow Emperor's Classics of Internal Medicine* that was published more than 2,000 years ago. The book states that the ear is related to all parts of the human body, particularly internal organs, and that all meridians converge at the ear [1]. The Chinese Association of Acupuncture and Moxibustion was tasked by the World Health Organization (WHO) to standardize acupoint locations to have a common language in the study and exchange of ideas [1]. The first document was accomplished in 1992 (GB/T 13734-1992) but was published in 1993 [2]. The latest nomenclature and location of auricular points announced by the China Standardization Organizing Committee in 2008 (GB/T 13734-2008) are more comprehensive because the committee has added additional information about the anatomical classification and location of the various parts of the auricle.

The reflexive property of the ear can cause various physical attributes to appear on the auricle in the presence of bodily disorders. These attributes include variations in shape, color, size, and sensation; the appearance of papules, creases, and edema; and increased tenderness or decreased electrical conductivity [3, 4]. Auricular signals associated with body functions have been reported in previous studies. Kwai-Ping Suen et al. [5] found that participants with coronary heart disease (CHD) exhibit the presence of earlobe crease compared with those without CHD. The “heart” zone of the CHD cases significantly showed higher conductivity and tenderness in both ears than those of the controls. Abnormal hair growth on the ear is a sign of hormonal changes that accompany the decline in kidney* qi*, which occurs with age [3]. Discoloration and marks after pressing that appears in the “pancreas and gallbladder” (P&G) region of the ear, as well as increased electrical resistance of this acupoint, may be detected in cases with diabetes mellitus (DM) [6, 7].

According to the auricular diagnosis system, areas of the auricle with increased electrical conductivity and heightened tenderness upon touching correspond to specific areas of the body where some pathological conditions exist [4, 8]. When a disease or disorder is present in the body, electrical resistance in the corresponding auricular points obviously decreases. Areas where electrical resistance is lower than the standard are also considered as positively or highly conductive electrical resistance points [1].

Over the past three decades, the number of people with DM has been more than doubled globally [9]. Type 2 DM (T2DM) and prediabetes are increasingly occurring among children, adolescents, and younger adults [10]. In addition, 30% of patients with renal failure, stroke, heart failure, and myocardial infarction have DM as a major contributory factor; 30% of patients are diagnosed with retinopathy at the time of referral [11]. Therefore, earlier screening of T2DM through a noninvasive and effective approach is necessary. Aside from the report on the relationship between auricular signals and T2DM [6, 7], no previous study has systematically examined these auricular signals through inspection, detection of electrical resistance, and tenderness testing on T2DM patients; most findings on auricular diagnosis were reported by case studies [6, 7]. The association between auricular signals and T2DM should be further investigated because auricular diagnosis is an objective, painless, and noninvasive method that provides rapid access to information. Such investigation would help extrapolate the research findings to the vulnerable population for purposes of DM risk assessment.

## 2. Aim and Hypotheses

This study aimed to investigate the association between auricular reflective points and the status of T2DM among the Chinese in Hong Kong. The hypotheses of this study are as follows. (1) During visual inspection, significant difference(s) in terms of discoloration and/or marks appearing after pressing is/are found in the appearance of the “P&G” region of the auricles between clients with or without T2DM. (2) Significant differences exist in tenderness on the “P&G” region of the auricles between participants with or without T2DM. (3) Significant differences exist in electrical skin resistance on selected auricular points between participants with or without T2DM. (4) The auricular signals on T2DM patients are positively associated with the years of DM diagnosis, glycemic control based on glycated hemoglobin (HbA1c) readings, and presence of T2DM complications (including macrovascular complications, such as cardiovascular diseases, and microvascular complications, such as retinopathy and chronic kidney disease stage 2 or above).

The selected auricular points related to T2DM include “P&G,” “endocrine,” “kidney,” “lower tragus,” “heart,” and “eyes.” Point selection was based on “organ” theory of Chinese medicine, as well as on principles of Western medicine. “P&G” and “endocrine” were selected because T2DM is attributed to insufficient insulin production by the pancreas and is an endocrine problem. “Kidney,” “heart,” and “eyes” were chosen because chronic kidney disease, cardiovascular problems, and retinopathy are common complications of T2DM, respectively [12–14]. The point of “lower tragus” is known as “*xiao ke*” in Chinese medicine, which means “diabetes point” [1]. Consultation by a registered TCM practitioner on points selection was also made.

## 3. Methods

### 3.1. Settings and Participants

A total of 282 participants were recruited in this matched case-control study. This group comprised 141 T2DM cases and an equal number of matched controls by age group and gender. Cases were defined as those diagnosed with T2DM and were recruited from the department of medicine and geriatrics of a regional hospital in Hong Kong. Controls were defined as those who do not have previous medical history of T2DM and were recruited from the community (e.g., carers of T2DM patients or those from centers of the elderly). Only subjects aged 40 years or above were eligible to participate. The age distribution is as follows: 40 to 49 (*n* = 10, 14%), 50 to 59 (*n* = 15, 21%), 60 to 69 (*n* = 20, 27%), 70 to 79 (*n* = 18, 26%), and 80 to 100 (*n* = 7, 10%). HbA1c testing (by Bayer's A1CNow+ test kits) [15] was performed in the controls to ensure that they do not have T2DM. HbA1c testing for DM diagnosis (with a threshold of ≥6.5%) is widely recognized by the American Diabetes Association (ADA), the International Diabetes Federation, and the European Association for the Study of Diabetes [12]. Subjects with aural injury, deformities, or infections that might affect the shape and crease of the ears were excluded from the study.

The existing treatment protocol for T2DM cases in the hospital under study is as follows. Lifestyle modification is the first step for controlling diabetes. If oral antidiabetic treatment is needed, metformin at 500 or 1000 mg daily (twice or thrice daily) is used as first-line management. If optimal glycemic control is not achieved, sulfonylurea group of medication will be added. Insulin therapy will be supplemented if two or more types of oral antidiabetic drugs (OADs) at maximum doses have been administered but the glycemic control remains unsatisfactory. Occasionally, the third type of OAD such as DPP4-inhibitor or glitazone may be added if the patient is reluctant to start insulin therapy. If supplementary insulin therapy fails to control blood glucose, full replacement with insulin therapy once or twice daily will then be considered.

### 3.2. Data Collection

HbA1c testing and a simple health assessment that covers the common risk factors of T2DM, including blood pressure, body mass index, and medical and family history, were conducted. Ear diagnosis was performed in three ways: inspection, electrical detection, and tenderness testing. The researcher involved in auricular examination was blinded to the grouping of subjects.

*(a) Visual Inspection.* Both auricles were observed for discoloration (present/absent) and marks after pressing (present/absent) in the “P&G” region. 

*(b) Tenderness Testing.* A pressure algometer (force gauge) with a unit range of 0 g to 500 g was used to apply force on the “P&G” region on both auricles using the “shenmen” as reference. The researcher recorded the observed value (*g*) when the subject started to feel a tender sensation when the pointer of the instrument was applied on the acupoints under testing. 

*(c) Electrical Skin Resistance Measurement.* An individual threshold was set for each subject before assessing the ear acupoints. An electrical acupoint detector (Pointer Plus) [16] was used to measure auricular electrical resistance in the selected acupoints of the auricles with the “shenmen” as reference.

Skin resistance may be affected by some factors, such as skin humidity level, constitution, weather, and skills for examination. Therefore, individual threshold using the reference point (shenmen) will be considered to compare the acupoints under testing. The reasons for selecting “shenmen” as a reference point are because it is usually reactive to the effects of everyday stress in a person's life and is consistently identified in most people [4]. Setting the threshold was performed by placing the acupoint detector on the “shenmen” and by increasing the detection sensitivity until sound, light, or visual meter on the equipment indicated high electrical conductance. The sensitivity was then reduced slightly until the “shenmen” was only barely picked up [1, 6]. During the testing, the skills were also closely observed. For example, moving the detector very quickly can easily miss a reactive ear point, whereas applying excessive pressure with the detector may create false ear points merely because of the increased electrical contact with the skin. A proper procedure for holding the detector perpendicular to the stretched surface of the ear and gently gliding the detector over the ear was performed. No ear cleaning should be done before testing. If the examination was conducted in cold weather, the client would rest for at least 10 min in room temperature before the procedure.

*Other Parameters.* The finding of HbA1c reading (within three months) for the DM group was retrieved to assess the glycemic control over the last three months. Glycemic control was categorized as follows: in control, ≤6.9%; for close monitoring, 7.0% to 8.4%; for elevated levels, 8.5% to 10.4%; and for seriously elevated levels, 10.5% and above. Information regarding the presence of macrovascular complications (such as CHD and stroke), microvascular complications (such as retinopathy and diabetic nephropathy), and other risk factors (such as smoking and central obesity) was also collected. Subjects were defined as having cardiovascular problems when they were diagnosed of having CHD or stroke. The presence of diabetic retinopathy was classified as (1) mild nonproliferative, (2) moderate nonproliferative, (3) severe nonproliferative, (4) very severe nonproliferative, and (5) clinically significant macular edema, to be determined by the ophthalmologist. Subjects were diagnosed of having nephropathy if the patient was classified as being of stage 3 of chronic kidney disease, with the GRF <60 mL/min/1.73 m^2^ [17].

## 4. Validity and Reliability

The interobserver agreement of observations by the principal investigator and the researcher involved in the data collection was recorded for 10 cases to assess the reliability of the auricular examinations, and an agreement of 92% was achieved. Both of them were involved in performing the auricular examination. If discrepancies in the assessment occur between the two raters, a consensus was sought after discussion. During the auricular examination, the data collection forms which contain the medical history of the subjects could not be accessed by the raters, so as to ensure blinding to the subjects grouping to avoid observer bias.

## 5. Data Analyses

The sensitivity, specificity, and positive and negative predictive values (PPV and NPV) of auricular signals (presence or absence of discoloration, marks after pressing, tenderness, and electrical resistance) of the auricular point(s) under testing were computed. McNemar test was used to determine the association between auricular signals and T2DM status (+ve versus −ve). Chi-square test was used to examine the association between auricular signals and occurrence of T2DM complications among the cases. Multivariate conditional logistic regression model was used to determine whether the T2DM status of the subjects is associated with auricular signals under testing. Analyses were conducted using IBM-SPSS Statistics 21. A *p* < 0.05 was considered statistically significant.

## 6. Ethical Considerations

Ethical approval from the study hospital and the university with which the first author is affiliated was sought. Written informed consent was obtained from all participants. The purpose and procedures of the study were explained verbally and in writing to the participants. Participation in the study was voluntary, and all participants were assured that they have the right to refuse or withdraw from the study at any time. Personal information and data remained confidential and anonymous.

## 7. Results

### 7.1. Demographic Characteristics and DM-Related Conditions

A total of 242 participants were recruited, among which 141 cases (T2DM +ve) were from a regional hospital in Hong Kong, and an equal number of controls (T2DM −ve) were from the community. The participants in the case and control groups were matched by age group and gender. The mean age of the participants was 63.91 years (SD = 12.70) with 141 males and 141 females. No significant gender distribution between the groups was observed. Participants in the case group had higher BMI (*p* < 0.001), waist-to-hip ratio (*p* < 0.001), and DBP (*p* < 0.01) as well as being more likely to smoke (*p* < 0.05) and to have comorbidity (*p* < 0.001) than those in the control group. Moreover, it was found that subjects with tenderness or electrical conductivity in the selected acupoints (“P&G,” “endocrine,” “kidney,” “lower tragus,” “heart,” and “eyes”) were found to have higher BMI, waist-to-hip ratio, and DBP. Therefore, these variables were adjusted for subsequent multivariate analyses. Among the T2DM cases, the average duration of diagnosis was 13.31 years (SD 8.38); 22.7% (*n* = 32) had cardiovascular problems, 36.6% (*n* = 45) had diabetic retinopathy, and 22.7% (*n* = 32) had nephropathy (Table 1).

### 7.2. Auricular Signals

The auricular signals that appeared on T2DM +ve patients were compared with those of T2DM −ve patients (Table 2). 

*(1) Visual Inspection.* All participants who showed presence of marks around the “P&G” region after pressing belonged to the T2DM +ve group (*n* = 6). An average of 4.3% of the participants in the T2DM +ve group had marks around the “P&G” region after pressing on the right ear, whereas none of the participants had marks in the T2DM −ve groups. No significant differences were observed for discoloration around the “P&G” region (*p* > 0.05). 

*(2) Tenderness Testing.* The participants in the T2DM +ve group experienced significant tenderness in the “P&G” region in both ears (*p* < 0.001) compared with those in the T2DM −ve group. The tenderness testing of the “P&G” region showed a sensitivity of 73.0%, a specificity of 70.2%, a PPV of 71.0%, and a NPV of 72.3% for the right ear and a sensitivity of 55.3%, a specificity of 68.1%, a PPV of 63.4%, and a NPV of 60.4% for the left ear (Table 3). 

*(3) Electrical Skin Resistance Measurement.* All the auricular points under testing (i.e., the “P&G,” “endocrine,” “kidney,” “lower tragus,” “heart,” and “eyes”) of the T2DM +ve cases had a significantly higher conductivity on both ears (i.e., less electrical skin resistance) than those of the control group (*p* < 0.01). Among these points, the conductivity of the “P&G” region exhibited the highest predictive power, with a sensitivity of 66.0%, a specificity of 73.8%, a PPV of 71.5%, and a NPV of 68.4% for the right ear and a sensitivity of 61.0%, a specificity of 75.2%, a PPV of 71.1%, and a NPV of 65.8% for the left ear (Table 3). 

*Multivariate Analyses.* Multivariate conditional logistic regression analysis was conducted to examine the association between tenderness and electrical conductivity on T2DM status after controlling for potential confounders (age, sex, smoking habit, alcohol consumption, MABP, BMI, and waist-to-hip ratio). The strongest association was demonstrated in the presence of electrical conductivity of the “P&G” region of the right ear (adjusted OR = 9.65, 95% CI = 3.14–29.60). The electrical conductivities at the “lower tragus” and “eyes” in both ears and the “kidney” in the left ear were significantly associated with T2DM status adjusted for age group and sex; however, association became insignificant after further adjustment for other potential confounders (Table 4). 

*Subgroup Analysis*. Among the T2DM +ve group, tenderness in the “P&G” region of the left ear and electrical conductivities at the “P&G” region of the left ear and at the “kidney” of the right ear were associated with glycemic control (HbA1c, ≤6.9% versus ≥7.0%) (*p* < 0.05). The duration of T2DM (<10 years versus ≥10 years) was also significantly associated with the tenderness in the “P&G” region of the left ear (*p* < 0.05). In addition, the electrical conductivity at the “P&G” region on the right side of the ear was associated with the presence of retinopathy (*p* < 0.05), and tenderness on the same region was associated with the occurrence of nephropathy (*p* < 0.05).

## 8. Discussion

The current case-control study adopted a systematic and scientific approach using visual inspection, tenderness testing, and electrical skin resistance measurement to investigate the auricular signals and their relationships with T2DM. French physician Dr. Paul Nogier hypothesized that the human body can be projected into the auricle in the same way of projection into the brain cortex [18]. This idea accords with* Lingshu Jing* (also known as* Divine Pivot*), an ancient Chinese medical text, where “the ears are the confluence of the channels.” This implies that the ears are related to the internal organs through the channels and collaterals and that they are directly or indirectly related to the 12 main pairs of meridians that run over the body [19]. Suen et al. [5] found that participants with CHD exhibit the presence of earlobe crease compared with those without CHD, and the “heart” zone of these cases significantly showed higher conductivity and tenderness in both ears than those of the controls.

In this study, no statistically significant differences were found in the discoloration of the “P&G” regions between cases and controls, but all participants who showed the presence of marks around the “P&G” region after pressing belonged to the T2DM +ve group. The participants in the T2DM group experienced significant tenderness in the “P&G” region compared with those in the controls as detected by the pressure algometer (force gauge). In the current study, the duration of T2DM was significantly associated with the tenderness of the “P&G” region on the left ear. This accords with previous studies where the degree of tenderness is usually related to the severity of the condition and a more sensitive point indicates a more severe disorder [4, 20]. Oleson [21] demonstrated in an animal experiment that the skin acupoints on dog bodies show significantly higher concentrations of substance P than the acupoints of the controls. Substance P is a spinal neurotransmitter found in afferent C-fibers involved in pain transmission. Therefore, an increase in substance P would decrease the pain threshold and would make the auricular points tender upon touching [21]. Among the three examination methods adopted in the current study, tenderness testing of the “P&G” region on the right ear exhibited the highest sensitivity (73.0%) for the prediction of T2DM status.

Some auricular points (including “P&G,” “endocrine,” “kidney,” “lower tragus,” “heart,” and “eyes”) under testing in the cases showed significantly higher skin conductance on both ears (i.e., less electrical skin resistance) than those in the control group. The electrical resistance in the corresponding auricular point decreases when a disease or disorder is present in the body; areas with low electrical resistance compared to the reference point are considered highly conductive electrical resistance points [1]. Several reasons may explain this phenomenon. From a physiological perspective, the changes in electrical resistance on the auricular points can be ascribed to a change in the electrical resistance on the underlying cell membranes [22]. The electrical resistance on the cell membranes would be lower when it is malfunction. The signal is then transmitted to the central nervous system through the meridians, which subsequently sends signals to the corresponding auricular points and changes the electrical resistance [22]. Oleson et al. [23] hypothesized that a somatotopic organization of the body is represented on the human auricle, and such alterations in skin conductivity at painful areas of the body are attributed to the regional hyperactivity of the sympathetic nervous system. Activation of sympathetic nerves may also cause a change in skin moisture level and may reduce electrical resistance [24]. The cases with poorer glycemic control were significantly associated with tenderness and conductivity of the “P&G” region on the left ear.

**Figure 1:**
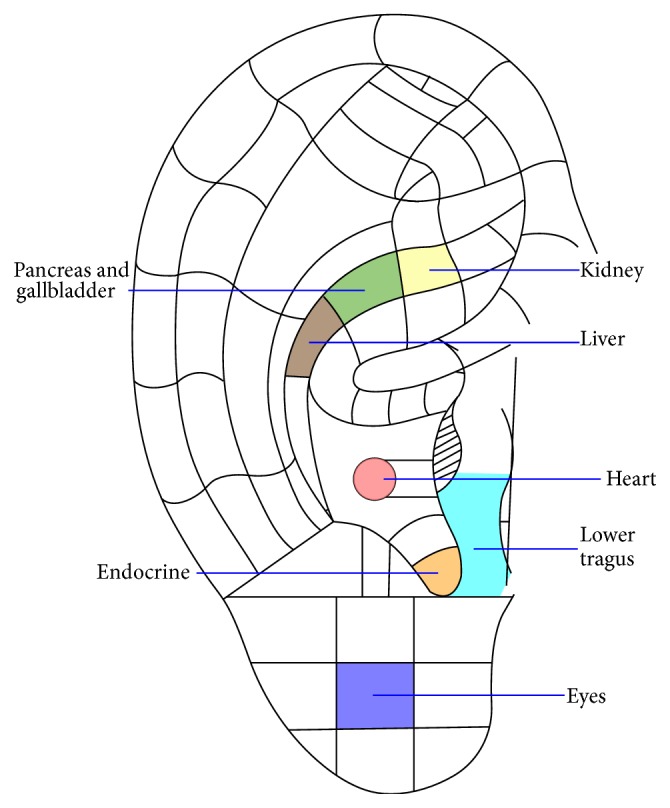


Though the ears are innervated by different nervous system/nerve endings, such as cervical nerves, temporal nerve, vagus nerve, and sympathetic nervous system [4], the principle for selecting the acupoints in the current study was not affected by their nature of parasympathetic and sympathetic dominance. The selection was ascribed to the underlying principles of either Chinese or Western medicine and to the association of these acupoints with T2DM. For example, DM is caused by insufficient insulin production of the pancreas and is an endocrine problem; thus, “P&G” and “endocrine” were selected. “Kidney,” “heart,” and “eyes” were chosen because nephropathy, cardiovascular problems, and retinopathy are common complications of T2DM, respectively [12–14]. The point of “lower tragus” is also known as “*xiao ke*” in Chinese medicine, meaning “diabetes point” [1] (Figure 1).

Interestingly, some acupoints were significantly found on either the right or left ear and sometimes on both ears. The pathway for developing auricular signals in T2DM cases is not clearly elucidated. Based on anatomical location, the head of the pancreas lies in the right upper quadrant and the body of the pancreas lies in the left upper quadrant. In 80% to 90% of the individuals, the reactive points of the ears are found on the same ear as the side of the body where the problem lies. For the other 10% to 20% of the cases, the presentation is contralateral, and the most reactive ear point is found on the opposite side of the body [4].

HbA1c has long been used in the management of established diabetes as a biomarker of glycemic control. The levels of this end product of nonenzymatic glycation in blood correlate well with average ambient blood glucose levels during the previous eight to twelve weeks. HbA1c measurement was subsequently adopted as an alternative method of DM diagnosis by the ADA and the WHO in 2010 and in 2011, respectively. Despite some advantages, the use of HbA1c testing has limitations. This method lacks reliability in patients with hemoglobinopathies, unreliability in certain anemia cases with high or low red-cell turnover, and lack of reliability after recent transfusion. The cost and availability of HbA1c would be a hindrance for the wide adoption of this measure as a diagnostic approach. In addition, the discordant diagnosis of DM using HbA1c and glucose criteria is of concern [10, 25].

Aside from the auricular examinations adopted in the current study, other auricular diagnostic methods were mentioned in previous literature. For example, vascular autonomic signal is related to a change in pulse amplitude or waveform of pulse in response to auricular stimulation [4, 26]. However, the mastery of such technique requires practice and training. Guan et al. [27] also proposed the use of auricular dyeing for diagnostic purpose. This method may not be convenient and may not be widely accepted by people under testing because the auricles will be dyed during the process. In comparison, the auricular diagnostic method in the current study is a simple, painless, and noninvasive method that provides rapid access to information.

Reflective points on the ears reflect the ongoing condition of a disease, indicating the present health condition as well as the status in the past and even near future. Positive auricular points may indicate when pathology is completely healed [4]. DM is a metabolic disease characterized by hyperglycemia resulting from progressive defects in insulin secretion and action. Chronic hyperglycemia is associated with long-term complications of different organs, particularly the eyes, kidneys, nerves, heart, and blood vessels [13, 28]. In the current study, the majority of the T2DM cases were associated with complications of different degrees. DM is usually silent in its initial stages, and irreversible complications may develop before treatment has begun [25]. Diabetic nephropathy is a common complication among patients with T2DM and is characterized by albuminuria (≥300 mg/d) and reduced glomerular filtration rate (<60 mL/min/1.73 m^2^). This complication often develops during the prediabetes stage and is present at the time of diabetes diagnosis. Conscientious screening will facilitate detection of kidney impairment earlier, when interventions to prevent or delay progression are more effective [29]. Retinopathy is identified in a group of patients who were younger yet had a longer known duration of DM with worse glycemic control and higher rates of insulin use [14]. The findings of the current study indicate that the longer duration of T2DM results in poorer glycemic control of cases, which are more prone to have tenderness and/or electrical conductivity of the “P&G” region on the left ear. Cases with retinopathy or nephropathy are also associated with the auricular signals of the ears to a certain extent. Therefore, auricular diagnosis could be considered as a screening method for vulnerable populations with T2DM risk, such as those with family history of DM, hypertension, hyperlipidemia, sedentary lifestyle, and obesity.

Aside from the diagnostic value of the acupoints under study, these acupoints have also been used for T2DM patients to achieve therapeutic effects. Auricular acupuncture is an adjuvant therapy that can regulate blood glucose and lipids and can decrease body weight in T2DM patients [30, 31]. Therefore, when reactive points are identified (e.g., when tenderness and/or electrical conductivity existed), stimulating these points can be considered using an appropriate method of auriculotherapy for therapeutic purpose.

## 9. Implications of Findings

Asia has gradually emerged as the “diabetes epicenter” in the world because of rapid economic development, urbanization, and nutrition transition over a relatively short period of time [10, 32]. DM is also an increasing health problem worldwide, which is projected to affect more than 400 million adults by the year 2030 [33]. T2DM is preceded by a lengthy asymptomatic stage, termed prediabetes, which is characterized by mild hyperglycemia, insulin resistance, and early decrements in insulin secretory capacity [25]. Prediabetes represents an elevation of plasma glucose above the normal range but below that of clinical diabetes and can carry some predictive abilities for macrovascular disease [34]. If the undiagnosed individuals/those with impaired glucose tolerance are left uncontrolled, they are subject to higher risks of developing diabetes and its complications. These situations will induce a negative effect both medically and financially [32]. Earlier identification of persons with prediabetes could allow for introduction of interventions to reduce risks [25].

The ear is a valuable tool in revealing constitutional predispositions. Auricular diagnostic method, if deemed effective, is a simple, effective, and inexpensive complementary approach that could integrate Chinese and Western models of care in screening T2DM status. The findings of the current study could advance our knowledge in understanding the association between specific auricular reflective signs and status of T2DM. Such information will be valuable background data to support future studies for screening vulnerable populations with T2DM risk, such as those with family history of DM, hypertension, hyperlipidemia, sedentary lifestyle, and obesity. Auricular diagnosis is speculated to have a prediagnostic value and to possess a role in the secondary level of prevention. If pre-DM status can be identified at an earlier stage, appropriate interventions, such as lifestyle change (diet and exercise), management of blood pressure and lipid levels, weight reduction, and physical activity, can be implemented to prevent or delay the progression of T2DM.

## 10. Limitations of the Study

Although the association between certain auricular points and T2DM is suggested in this study, the mechanisms leading to the development and the time of onset of these signals remain uncertain. A prospective cohort study should be conducted in the future to follow up vulnerable populations with DM risk or cases with prediabetes and to identify changes in the auricular signals that may have appeared during disease progression. Even with the use of constant pressure, maintaining a consistent placement of the probe against the external ear of the subjects is not always easy, thus leading to unreliable findings [4]. This study was conducted on a sample limited to one regional hospital. Therefore, further investigations must be performed with a larger sample to validate the use of auricular signals as predictors that assist in T2DM diagnosis. The prevalence of auricular signals may be governed by ethnical differences; thus, more work is required before these results can be extrapolated to other ethnic groups.

## 11. Conclusion

The results of this study suggest that the tenderness and electrical conductivity of a number of auricular points, including “pancreas and gallbladder,” “endocrine,” “kidney,” “lower tragus,” “heart,” and “eyes,” were associated with T2DM status in the Chinese population. In the subgroup analyses, certain auricular signals were also associated with glycemic control, duration of disease, and related complications. Auricular diagnosis could be considered as a screening method for vulnerable populations with T2DM risk; thus, appropriate interventions can be implemented to prevent or delay the progression of T2DM.

## Figures and Tables

**Table 1:** Sociodemographic data and DM-related conditions of participants.

	DM +ve (*n* = 141)	DM −ve (*n* = 141)	Test statistics (McNemar test)
Age (years)	64.21 (12.05)	63.62 (13.35)	*t* = 1.27 *p* = 0.21
Gender			N/A
Male	70 (49.6%)	70 (49.6%)	
Female	71 (50.4%)	71 (50.4%)	
BMI (kg/m^2^)	26.31 (4.29)	23.06 (3.14)	*t* = 7.50 *p* < 0.001^∗^
Waist-to-hip ratio	0.96 (0.11)	0.86 (0.07)	*t* = 9.27 *p* < 0.001^∗^
Comorbid illness			*χ* ^2^ = 15.61 *p* < 0.001^∗^
Yes	135 (95.7%)	112 (79.4%)	
No	6 (4.3%)	29 (20.6%)	
Smoking status			*χ* ^2^ = 9.54 *p* < 0.05^∗^
Never	97 (68.8%)	114 (80.9%)	
Given up	32 (22.7%)	20 (14.2%)	
Occasionally	4 (2.8%)	1 (0.7%)	
Regularly	8 (5.7%)	6 (4.3%)	
Alcohol consumption			*χ* ^2^ = 9.74 *p* = 0.08
Never	84 (59.6%)	102 (72.3%)	
Given up	16 (11.3%)	11 (7.8%)	
Occasionally	38 (27.0%)	26 (18.4%)	
Regularly	3 (2.1%)	2 (1.4%)	
Mean arterial pressure (MABP): diastolic BP + (systolic − diastolic BP)/3	97.78 (12.74)	94.67 (11.86)	*t* = 1.97 *p* = 0.051
Systolic blood pressure (mmHg)	137.07 (22.60)	135.19 (19.89)	*t* = 0.68 *p* = 0.50
Diastolic blood pressure (mmHg)	78.13 (10.61)	74.42 (10.90)	*t* = 3.01 *p* < 0.05^∗^
Family history of DM			
Yes	67 (47.5%)		
No	74 (52.5%)		
Duration of illness (years) Mean (SD)	13.31 (8.38)		
Presence of CV problem, including coronary heart disease and stroke			
Yes	32 (22.7%)	9 (6.40%)	
No	109 (77.3%)	132 (93.6%)	
Presence of diabetic retinopathy			
Yes	45 (36.6%)		
No	78 (63.4%)		
(i) Mild nonproliferative	38 (82.6%)		
(ii) Moderate nonproliferative	3 (6.5%)		
(iii) Severe nonproliferative	1 (0.7%)		
(iv) Very severe nonproliferative	0 (0.0%)		
(v) Clinically significant macular edema	1 (0.7%)		
Presence of nephropathy			
Yes	32 (22.7%)		
No	109 (77.3%)		

^∗^Statistically significant.

**Table 2:** Auricular signals between cases and controls.

	DM +ve (*n* = 141)	DM −ve (*n* = 141)	Test statistics (McNemar test)
Discoloration around “P&G” region (Rt)			
Yes	1 (0.7%)	0 (0.0%)	N/A
No	140 (99.3%)	141 (100.0%)	
Discoloration around “P&G” region (Lt)			
Yes	1 (0.7%)	0 (0.0%)	N/A
No	140 (99.3%)	141 (100.0%)	
Marks appearing around “P&G” region after pressing (Rt)			
Yes	6 (4.3%)	0 (0.0%)	N/A
No	135 (95.7%)	141 (100.0%)	
Marks appearing around “P&G” region after pressing (Lt)			
Yes	2 (1.4%)	0 (0.0%)	N/A
No	139 (98.6%)	141 (100.0%)	
Tenderness (P&G) (Rt)			*χ* ^2^ = 44.44 *p* < 0.001^∗^
Positive	103 (73.0%)	42 (29.8%)	
Negative	38 (27.0%)	99 (70.2%)	
Tenderness (P&G) (Lt)			*χ* ^2^ = 13.65 *p* < 0.001^∗^
Positive	78 (55.3%)	45 (31.9%)	
Negative	63 (44.7%)	96 (68.1%)	
Conductivity (P&G) (Rt)			*χ* ^2^ = 38.78 *p* < 0.001^∗^
Positive	93 (66.0%)	37 (26.2%)	
Negative	48 (34.0%)	104 (73.8%)	
Conductivity (P&G) (Lt)			*χ* ^2^ = 36.23 *p* < 0.001^∗^
Positive	86 (61.0%)	35 (24.8%)	
Negative	55 (39.0%)	106 (75.2%)	
Conductivity (endocrine) (Rt)			*χ* ^2^ = 20.74 *p* < 0.001^∗^
Positive	88 (62.4%)	50 (35.5%)	
Negative	53 (37.6%)	91 (64.5%)	
Conductivity (endocrine) (Lt)			*χ* ^2^ = 20.42 *p* < 0.001^∗^
Positive	73 (51.8%)	37 (26.2%)	
Negative	68 (48.2%)	104 (73.8%)	
Conductivity (kidney) (Rt)			*χ* ^2^ = 22.12 *p* < 0.001^∗^
Positive	83 (58.9%)	41 (29.1%)	
Negative	58 (41.1%)	100 (70.9%)	
Conductivity (kidney) (Lt)			*χ* ^2^ = 9.19 *p* < 0.001^∗^
Positive	67 (47.5%)	41 (29.1%)	
Negative	74 (52.5%)	100 (70.9%)	
Conductivity (lower tragus) (Rt)			*χ* ^2^ = 5.64 *p* < 0.001^∗^
Positive	61 (43.3%)	41 (29.1%)	
Negative	80 (56.7%)	100 (70.9%)	
Conductivity (lower tragus) (Lt)			*χ* ^2^ = 4.49 *p* < 0.001^∗^
Positive	54 (38.3%)	37 (26.2%)	
Negative	87 (61.7%)	104 (73.8%)	
Conductivity (heart) (Rt)			*χ* ^2^ = 19.76 *p* < 0.001^∗^
Positive	80 (56.7%)	44 (31.2%)	
Negative	61 (43.3%)	97 (68.8%)	
Conductivity (heart) (Lt)			*χ* ^2^ = 21.39 *p* < 0.001^∗^
Positive	74 (52.5%)	36 (25.5%)	
Negative	67 (47.5%)	105 (74.5%)	
Conductivity (eyes) (Rt)			*χ* ^2^ = 7.78 *p* < 0.05^∗^
Positive	78 (55.3%)	54 (38.3%)	
Negative	63 (44.7%)	87 (61.7%)	
Conductivity (eyes) (Lt)			*χ* ^2^ = 6.78 *p* < 0.05^∗^
Positive	64 (45.4%)	43 (30.5%)	
Negative	77 (54.6%)	98 (69.5%)	

^∗^Statistically significant.

**Table 3:** Predictive power of various ear acupoints on the risk of DM.

	Sensitivity (%)	Specificity (%)	Positive predictive value (%)	Negative predictive value (%)
Marks appearing around “P&G” region after pressing, (Rt)	0.7	100	100	50.2
Marks appearing around “P&G” region after pressing, (Lt)	0.7	100	100	50.2
Tenderness, (Rt) (P&G)	73.0	70.2	71.0	72.3
Tenderness, (Lt) (P&G)	55.3	68.1	63.4	60.4
Electrical conductivity, (Rt)				
P&G	66.0	73.8	71.5	68.4
Endocrine	62.4	64.5	63.8	63.2
Kidney	58.9	70.9	66.9	63.3
Lower tragus	43.3	70.9	59.8	55.6
Heart	56.7	68.8	64.5	61.4
Liver	27.7	80.9	59.1	52.8
Eyes	55.3	61.7	59.1	58.0
Electrical conductivity, (Lt)				
P&G	61.0	75.2	71.1	65.8
Endocrine	51.8	73.8	66.4	60.5
Kidney	47.5	70.9	62.0	57.5
Lower tragus	38.3	73.8	59.3	54.5
Heart	52.5	74.5	67.3	61.0
Liver	25.5	83.7	61.0	52.9
Eyes	45.4	69.5	59.8	56.0

Sensitivity (true positive) = number of true positives/number of true positives + number of false negatives.

Specificity (true negative) = number of true negatives/number of true negatives + number of false positives.

Positive predictive value = number of true positives/number of true positives + number of false positives.

Negative predictive value = number of true negatives/number of true negatives + number of false negatives.

**Table 4:** Odds ratio of various ear acupoints on the risk of DM.

	Odds ratio^a^	95% CI	Odds ratio^b^	95% CI
Tenderness, (Rt) (P&G)	7.10^∗∗∗^	(3.66, 13.77)	6.74^∗∗∗^	(2.38, 19.08)
Tenderness, (Lt) (P&G)	2.57^∗∗∗^	(1.55, 4.26)	1.62	(0.75, 3.47)
Electrical conductivity, (Rt)				
P&G	6.09^∗∗∗^	(3.22, 11.52)	9.65^∗∗∗^	(3.14, 29.60)
Endocrine	3.71^∗∗∗^	(2.06, 6.70)	3.70^∗^	(1.32, 10.25)
Kidney	3.47^∗∗∗^	(2.02, 5.95)	3.06^∗∗^	(1.31, 7.15)
Lower tragus	1.91^∗^	(1.14, 3.20)	1.72	(0.74, 4.00)
Heart	3.77^∗∗∗^	(2.05, 6.95)	3.69^∗^	(1.26, 10.77)
Liver	1.67	(0.93, 2.99)	1.57	(0.59, 4.13)
Eyes	2.09^∗∗^	(1.26, 3.48)	1.94	(0.81, 4.66)
Electrical conductivity, (Lt)				
P&G	6.67^∗∗∗^	(3.31, 13.43)	2.82^∗^	(1.12, 7.09)
Endocrine	4.00^∗∗∗^	(1.14, 3.10)	2.74^∗^	(1.01, 7.41)
Kidney	2.24^∗∗^	(1.34, 3.74)	1.42	(0.63, 3.19)
Lower tragus	1.85^∗^	(1.07, 3.19)	1.48	(0.62, 3.54)
Heart	3.92^∗∗∗^	(2.13, 7.21)	3.34^∗^	(1.23, 9.08)
Liver	1.81	(0.98, 3.34)	2.25	(0.74, 6.81)
Eyes	2.11^∗∗^	(1.21, 3.64)	1.95	(0.73, 5.20)

^a^Adjusted for age and sex.

^b^Further adjusted for smoking habit, alcohol consumption, MABP, BMI, and waist-to-hip ratio.

^∗^Statistically significant at 5% level.

^∗∗^Statistically significant at 1% level.

^∗∗∗^Statistically significant at 0.1% level.

## Abbreviations

DM: Diabetes mellitus
T2DM: Type 2 diabetes mellitus
CHD: Coronary heart disease
OADs: Oral antidiabetic drugs
HbA1c: Glycated hemoglobin
PPV: Positive predictive value
NPV: Negative predictive value
P&G: Pancreas and gallbladder.
